# Detection of phosphorylated Akt and MAPK in cell culture assays

**DOI:** 10.1016/j.mex.2016.04.009

**Published:** 2016-04-29

**Authors:** Simon Molgaard, Maj Ulrichsen, Ditte Olsen, Simon Glerup

**Affiliations:** The Lundbeck Foundation Research Center MIND, Department of Biomedicine, Aarhus University, 8000C Aarhus, Denmark

**Keywords:** Immunocytochemistry followed by confocal microscopy and antibody validation, HEK 293 cells, Hippocampal neuron culture, Fluorescence, Confocal microscopy, Signalling, BDNF, Insulin

## Abstract

This article describes an immunocytochemistry (ICC) method for staining against phosphorylated forms of the kinases Akt (pAkt) and MAPK (pMAPK). Phosphorylation is induced upon their activation by a number stimuli including insulin and brain-derived neurotrophic factor (BDNF), and is prerequisite for a number of cellular processes including cell proliferation and survival [1], [2], [3], [4], [5], [6]. ICC using antibodies raised against specific phosphorylation sites allows cell-type specific and subcellular monitoring of kinase activation. Here, we test how four different antibodies against pAkt and pMAPK, respectively perform in different cell types following insulin or BDNF stimulation using different protocol conditions. We find that phospho-specific-antibodies generally perform better when using Triton X-100 as a permeabilization agent compared to Saponin. In addition, two antibodies against pAkt and two against pMAPK gave a clear increase in signal in cells stimulated with insulin or BDNF compared to the signal obtained in unstimulated cells. These antibodies also performed well when tested with western blotting. Our results illustrate that both the choice of antibody as well as protocol details are critical parameters for successful detection of phosphorylated forms of kinases by ICC. This article includes:

• A protocol for subcellular detection of phosphorylated Akt and MAPK.
• Validation of 8 antibodies by immunocytochemistry.
• Confirmation by western blotting

## Graphical abstract

**Activation of intracellular kinases upon BDNF-stimulation of cultured hippocampal neurons A**. Non-stimulated negative control. **B**. BDNF-stimulation of hippocampal neurons induces phosphorylation of Akt, and specific antibodies against such allows for visualization of phosphorylated Akt (pAkt) on a subcellular level. Here only shown for pAkt, however, the paper also discuss visualization of phosphorylated MAPK.

**Figure:**
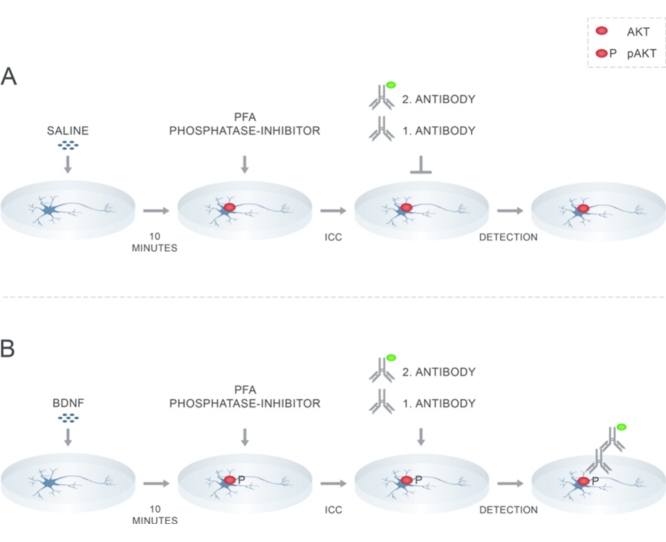

## Method details

The ICC protocol presented in this paper is designed to evaluate the specificity of antibodies against pAkt or pMAPK in HEK 293 cells and primary hippocampal neurons, stimulated with different concentrations of insulin or BDNF, respectively. The specificity of the antibodies was evaluated with respect to two different permeabilization agents, Triton X-100 or saponin, as the choice of permeabilization agent is important for the ability of the antibody to penetrate the cell membrane and bind to its target(s).

Four different antibodies against pAkt and four different antibodies against pMAPK were tested, and stimulated cells were compared to non-stimulated controls. All antibodies were rated on the independent antibody-review site www.pAbmAbs.com according to their performance.

The protocol includes four steps: Step 1: preparation of cell cultures, Step 2: Stimulation and fixation of cultured HEK 293 cells and primary hippocampal neurons, Step 3: Immunocytochemistry of HEK 293 cells and hippocampal neurons and confocal microscopy and finally Step 4: Evaluation of antibody specificity. Moreover, the specificity of the antibodies was confirmed by western blotting.

## Step 1: cell cultures

### HEK 293 cells

*Materials:*

• *HEK 293 cell medium*:

DMEM (Lonza, # BE12-604F/U1) supplemented with 10% Fetal bovine serum (FBS; Gibco, #10270-106) and Penicillin-Streptomycin (Gibco, #15140)

• 2.5% Trypsin (Gibco, #15090-046)
• 0.2 M EDTA (Sigma, #03690)
• HBSS: Hanks balanced salt solution (Life Technologies, #14025-092)

*Procedure*:

1. The HEK 293 cells were maintained in HEK 293 cell medium, until they reached a confluence of 100%.
2. At 100% confluence, the cells were dissociated from the culture well by removing the cell medium and adding 0.25% trypsin and 0.2 M EDTA diluted in HBSS.
3. After one minute of incubation, HEK 293 cell medium was added and the cells resuspended.
4. The resuspended cells were seeded at a density of 50% in a 24 well culture tray in 1 mL HEK 293 medium and maintained at 5% CO_2_ and 37 °C until reaching a density of 70%.

### Primary hippocampal neuron culture

*Materials*:

*Note: This list includes only non-standard items. Common laboratory equipment is assumed to be available.*

• Postnatal day 0 (P0) C57BL/6j BomTac mouse pups
• Acid washed coverslips pre-treated for 24 h in 95% nitric acid and subsequently washed three times in ddH_2_O and stored in 96% ethanol
• Poly-d-Lysine (Sigma, #P7886)
• Mouse laminin (Life Technologies, #23017015–1 mg)
• Dulbecco’s Phosphate-Buffered Saline (D-PBS; Gibco, #14190-094)
• Leibowitz L-15 Medium (Life Technologies, #11415-049)
• Papain (Bionordika, #WBT-LS003126)
• EDTA (Sigma–Aldrich, #03690)
• NaOH
• DMEM (Lonza, #BE12-604F/U1)
• FBS (Gibco, #10270-106)
• DNAse1 (Sigma, #DN25)
• *Hippocampal neuron medium*: Neurobasal-A Medium (Life Technologies, #10888-022) supplemented with B-27 Supplement (Life Technologies, #17504-044), 20 nM FUDR (Sigma, #F0503), 20 nM Uridine (Sigma, #U3750), 2 mM GlutaMAX supplement (Gibco, #35050) and 1 mg/mL primocin (Invivogen, #ant-pm-2)

*Procedure*:

1. The day before the experiment, acid washed coverslips were incubated in 0.1 mg/mL Poly-d Lysine at 37 °C. After four hours, the coverslips were washed four times in sterile water, air dried and stored sterile at −20 °C until use.
2. Prior to the experiment, the Poly-d-Lysine coated coverslips were incubated for at least two hours in 20 μg/mL laminin.
3. Newborn pups (P0) were sacrificed by decapitation. The brains were removed and placed into a sterile Petri dish containing ice-cold D-PBS.
4. After removal of the meninges, the hippocampus was isolated and moved to a 15 mL Falcon tube containing ice-cold L-15 medium. The tube was kept on ice throughout this process.
5. The papain solution was prepared by adding 40 μL EDTA and 20 U/mL papain to 10 mL pre-warmed L-15 medium. After 15 min activation at 37 °C, 60 μL 0.2 M NaOH was added to adjust pH.
6. To sediment isolated hippocampal tissue, the tube containing the hippocampi was spun at 1200 rpm (on a Rotofix 32 rotor).
7. The supernatant was removed and the papain solution was sterile filtered into the 15 mL tube containing the hippocampi. The samples were then incubated for thirty minutes at 37 °C to digest the tissue.
8. The digestion was stopped by adding 5 mL DMEM containing 10% FBS and 5 μL DNAse1. The samples were hereafter spun at 1200 rpm to sediment tissue.
9. After removal of the supernatant, 1 mL DMEM containing 10% FBS and 1 μL DNAse1 was added, followed by a careful trituration using a P1000 tip until the tissue was completely dissociated.
10. Following this, 10 mL DMEM containing 10% FBS was added and the sample was centrifuged at 1200 rpm for five minutes before discarding the supernatant.
11. Neuron medium containing 1 μL DNAse1, pre-warmed to 37 °C, was added and the cells were resuspended.
12. Excess laminin was removed from the coverslips.
13. The cells were counted and seeded at a density of 100,000 cells per coverslip. The medium was then supplemented to a final volume of 1 mL hippocampal neuron medium per 24-well.
14. The cells were incubated at 37 °C in a humidified incubator with 5% CO_2_ for fourteen days. 0.5 mL of the hippocampal neuron medium was changed every second day.

### Step 2: stimulation and fixation of cultured HEK 293 cells and primary hippocampal neurons

*Materials*:

• HEK 293 cells and hippocampal neurons (fourteen *days in vitro*) from *Step 1*
• HEK 293 cell medium (*Step 1, HEK 293 cell culture, Materials*)
• Insulin (Sigma #I188)
• Hippocampal neuron medium (*Step 1, Primary hippocampal neuron culture, Materials*)
• BDNF (Millipore, #gf029)
• D-PBS (Gibco, #14190-094)
• 4% w/v paraformaldehyde (PFA; Sigma–Aldrich # P6148) in D-PBS, pH 7.4
• PhosStop (Roche, #04906837001)
• Sodium azide 10% w/v (Ampliqon, #AMPQ52300.050)

*Procedure*:

1. HEK 293 cells were used when reaching a density of 70%. Hippocampal neurons were used after 14 days in culture. At this point, the neurons have developed a complex dendritic tree.
2. The HEK 293 medium was removed and replaced with 0.5 mL HEK 293 medium containing 1, 10 or 100 nM insulin. For stimulation of cultured hippocampal neurons, the medium was replaced by neuron medium containing 1 or 10 nM BDNF. As control, both HEK 293 and hippocampal neurons were stimulated with media containing sterile D-PBS as control. The cultures were then incubated for ten minutes at 37 °C in a humidified incubator with 5% CO_2_.
3. After the ten minutes of incubation, the cells were placed directly on ice to decrease the dephosphorylation rate catalysed by phosphatases. The medium was removed and the cells were washed once in ice-cold D-PBS before adding 0.5 mL of ice-cold 4% PFA containing PhosStop to prevent dephosphorylation during fixation.
  *Note: It is critical to prevent dephosphorylation of phosphorylated proteins during the fixation step in order to be able to detect phosphorylation by immunostainings.*
4. The cells were fixed for twenty minutes on ice before a three times five minutes wash in D-PBS. The last washing step includes adding 0.01% sodium azide to prevent bacterial contamination, and the fixed cells were hereafter stored at 4 °C until use.

### Step 3: immunocytochemistry of HEK 293 cells and hippocampal neurons and confocal microscopy

*Materials*:

• Fixed HEK 293 cells and hippocampal neurons from *Step 2*
• D-PBS (Gibco, #14190-094)
• Triton X-100 (Applichem, #A1388)
• Saponin (Sigma, #84510)
• FBS (Gibco, #10270-106)
• Primary antibodies (Table 1)
• Alexa Fluor^®^ 488 Donkey Anti-Rabbit (Molecular Probes, #A-21206)
• Hoechst (Sigma, #861405)

*Procedure*:

*Permeabilization using Saponin as the detergent*:

1. The fixed HEK 293 cells were washed briefly in D-PBS to remove the 0.01% Sodium Azide.
2. The cells were permeabilized by washing 3 × 5 minutes in D-PBS containing 0.1% saponin, at room temperature (RT).
3. After a brief wash in D-PBS, the cells were incubated in 0.5 mL D-PBS containing 10% FBS and 0.1% saponin (Blocking buffer) for thirty minutes at RT. This step is to reduce unspecific binding of the antibodies.
4. After this, coverslips were incubated with primary antibody (Table 1), diluted in Blocking buffer overnight at 4 °C in a humidified chamber.
5. Before continuing the next morning, the coverslips were placed at RT for one hour to enhance antibody-antigen binding.
6. To remove the excess primary antibody, the cells were washed 3 × 5 minutes in D-PBS containing 0.1% saponin.
7. The cells were then incubated with Alexa Fluor^®^ 488 Donkey Anti-Rabbit IgG, diluted 1:300 in Blocking buffer, in a dark humidified chamber at RT for four hours.
8. The cells were then washed 3 × 5 minutes in D-PBS, and 5 μg/mL Hoechst nuclear staining was included in the last wash.
9. The coverslips were mounted using Dako Fluorescence Mounting medium (Dako, S3023) and stored at 4 °C.
10. Finally, the immunostainings were analysed on a LSM 780 confocal microscope (Carl Zeiss) using the 63X/1.20 W Korr (Water immersion correction ring) objective.

*Permeabilization using Triton X-100 as the detergent*:

1. The fixed HEK 293 cells or hippocampal neurons were washed briefly in D-PBS to remove the 0.01% Sodium Azide.
2. The cells were permeabilized by washing 3 × 5 minutes in D-PBS containing 0.1% Triton X-100, at RT.
3. After this, the coverslips were washed once in D-PBS.
4. Unspecific binding of the antibodies was reduced by incubating the cells with 0.5 mL D-PBS containing 10% FBS (Blocking buffer) for thirty minutes at RT.
5. After this, the antibodies were incubated with primary antibody (Table 1), diluted in Blocking buffer, overnight at 4 °C in a humidified chamber.
6. Before continuing the next morning, the coverslips were placed at RT for one hour to enhance antibody-antigen binding.
7. To remove the primary antibody, the coverslips were washed 3 × 5 minutes in D-PBS containing 0.1% Triton X-100.
8. The cells were then incubated with Alexa Fluor^®^ 488 Donkey Anti-Rabbit IgG, diluted 1:300 in Blocking buffer, in a dark humidified chamber at RT for four hours.
9. The cells were then washed 3 × 5 minutes in D-PBS, and 5 μg/mL Hoechst nuclear staining was included in the last wash.
10. The coverslips were mounted using Dako Fluorescence Mounting medium (Dako, S3023) and stored at 4 °C.
11. Finally, the immunostainings were analysed on a LSM 780 confocal microscope (Carl Zeiss) using the 63X/1.20 W Korr (Water immersion correction ring) objective.

### Western blot analysis

Western blotting was performed to confirm the specificity of the antibodies.

For western blot analysis, the HEK 293 cells and primary hippocampal neurons were prepared and stimulated as described above (step 1 and 2 in the immunocytochemistry section). The only differences were that the cells were seeded in wells, which had been poly-l-lysine coated instead of seeding the cells on coverslips and the cells were only stimulated with one concentration of insulin (10 nM) or BDNF (1 nM).

*Materials*:

*Note: This list includes only non-standard items. Common laboratory equipment is assumed to be available*

• HEK 293 cells and primary hippocampal neurons
• Cell lysis buffer:

TNE buffer (10 mM Tris, 150 mM NaCl, 1 mM EDTA, 1% Igepal Ca 630 (Sigma-Aldrich), pH 8) supplemented with protease inhibitors (Complete)(Roche, # 000000011697498001)

• Bicinchonic Acid kit (Sigma-Aldrich, #BCA1)
• 4x LDS Sample buffer (Fisher Scientific, # NP0007)
• MOPS buffer (Sigma-Aldrich, #PCG3003)
• 20 mM dithiothreitol (DTT)(Applichem, # A1101,0005)
• Blocking buffer:

TST buffer (0.05 M Tris-Base, 0.5 M NaCl, and 0.1% (w/v) Tween-20) supplemented with 2% (w/v) skimmed milk powder

• Binding buffer:

MB buffer (2 nM CaCl_2_, 1 mM MgCl_2_, 10 mM HEPES, 140 mM NaCl, pH 7.8) containing 0.2% (w/v) skimmed milk powder and 0.05% Tween-20

• Strip buffer:

62.5 mM Tris–HCl, 100 mM 2-mercaptoethanol and 2% SDS

• Primary and secondary antibodies

*Procedure*:

1. The cells were lysed in lysis buffer for 10 minutes on ice.
2. Cells were scraped off with a cell scraper, and cell lysis were collected in eppendorf tubes.
3. The lysates were spun down at 20,800 rcf for 10 minutes.
4. The supernatant were transferred to new eppendorf tubes.
5. The protein concentration of the cells lysates were determined using a Bicinchonic Acid kit.
6. Samples were prepared by mixing appropriate amount of protein with NuPAGE LDS Sample Buffer and dithiothreitol (DTT, to a final concentration of 20 mM).
7. Samples were boiled for 5 minutes at 95 °C.
8. The samples were loaded onto a SDS denaturing gel (NuPAGE, 4–12% Bis–Tris polyacrylamide gel, Invitrogen) and run for 40 minutes (constant 200 V resulting in a current between 70 and 125 mA).
9. Proteins were transferred to nitrocellulose membranes by iBlot Gel Transfer Stacks (Invitrogen) according to manufacturer’s protocol (program 3, 20 V, 8 minutes).
10. The membranes were blocked by incubating with blocking buffer for 1 h on an orbital shaker.
11. Membranes were washed briefly and incubated with primary antibodies (anti-pMAPK antibodies (#9101 and #4370, Cell Signaling) or anti-pAkt antibodies (#2965 and #4060, Cell Signaling)) overnight at 4 °C on a tilting table.
12. Next morning, the membranes were washed 3 × 6 minutes in binding buffer on an orbital shaker.
13. The membranes were hereafter incubated with HRP-conjugated secondary antibodies (swine anti-rabbit, P0217 from Dako, 1:2000) for one hour at RT on an orbital shaker.
14. After 3 × 6 minutes washing in binding buffer on an orbital shaker, the proteins were visualized with ECL Western blotting detection system (GE Healthcare) and Fuji film LAS4000.
15. After development, the membranes were stripped by incubation in strip buffer for 20 minutes at 50 °C while rotating.
16. Membranes were washed briefly in binding buffer on an orbital shaker.
17. Membranes were incubated with antibodies recognizing total amount of Akt (#9272, Cell Signaling) and MAPK (#4695, Cell Signaling) overnight at 4 °C on a tilting table.
18. Next day the membranes were washed, incubated with secondary antibodies (swine anti-rabbit, P0217 from DAKO, 1:2000), washed again and developed as described above.

### Step 4: evaluation of the antibodies

Immunostaining against phosphorylated proteins can be a great challenge as it requires high specificity of the antibodies, i.e. that they only recognize the protein in question when it is phosphorylated. Akt and MAPK are phosphorylated in HEK 293 cells stimulated with insulin or primary hippocampal neurons stimulated with BDNF. This protocol describes a way to evaluate antibody specificity by comparing ICC stainings of stimulated cells to unstimulated negative controls, since a high level of staining indicates that the antibody recognizes a phosphorylated motif in the stimulated cells. To ensure that the antibody binds specifically to a certain phosphorylated motif, mutagenesis studies should be performed. This is, however, outside the scope of this protocol. Here we tested antibodies raised against specific phosphorylation sites on Akt and MAPK which are involved in numerous processes including survival, proliferation, differentiation and motility.

**Fig. 1:**
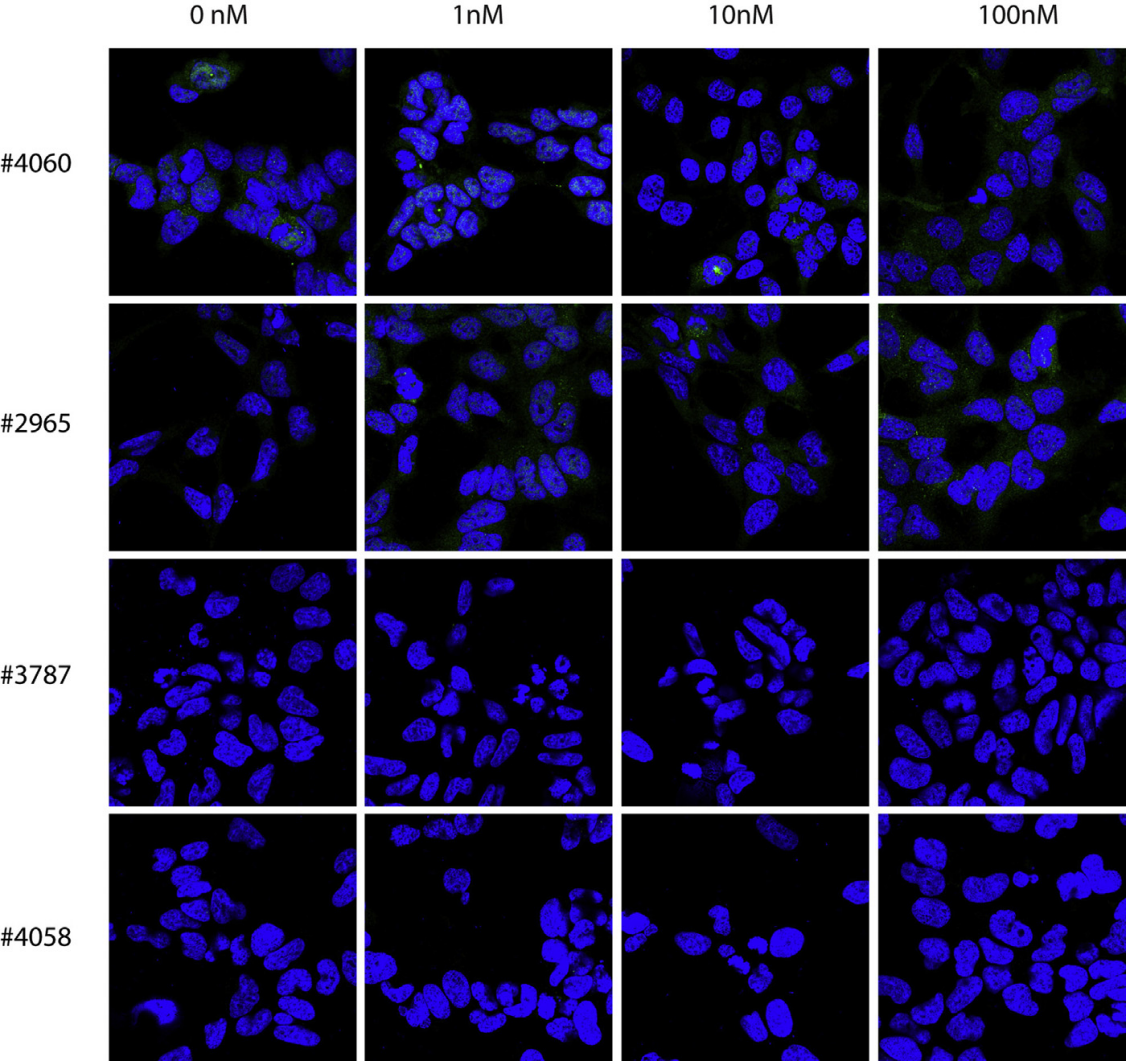
Immunocytochemical stainings against pAkt of 0, 1, 10 or 100 nM insulin-stimulated HEK 293 cells permeabilized with saponin. Four different antibodies against pAkt (#4060, #2965, #3787, #4058) were tested in three independent experiments, but none of the antibodies showed strong insulin-induced staining when saponin was used as the permeabilizing agent.

**Fig. 2:**
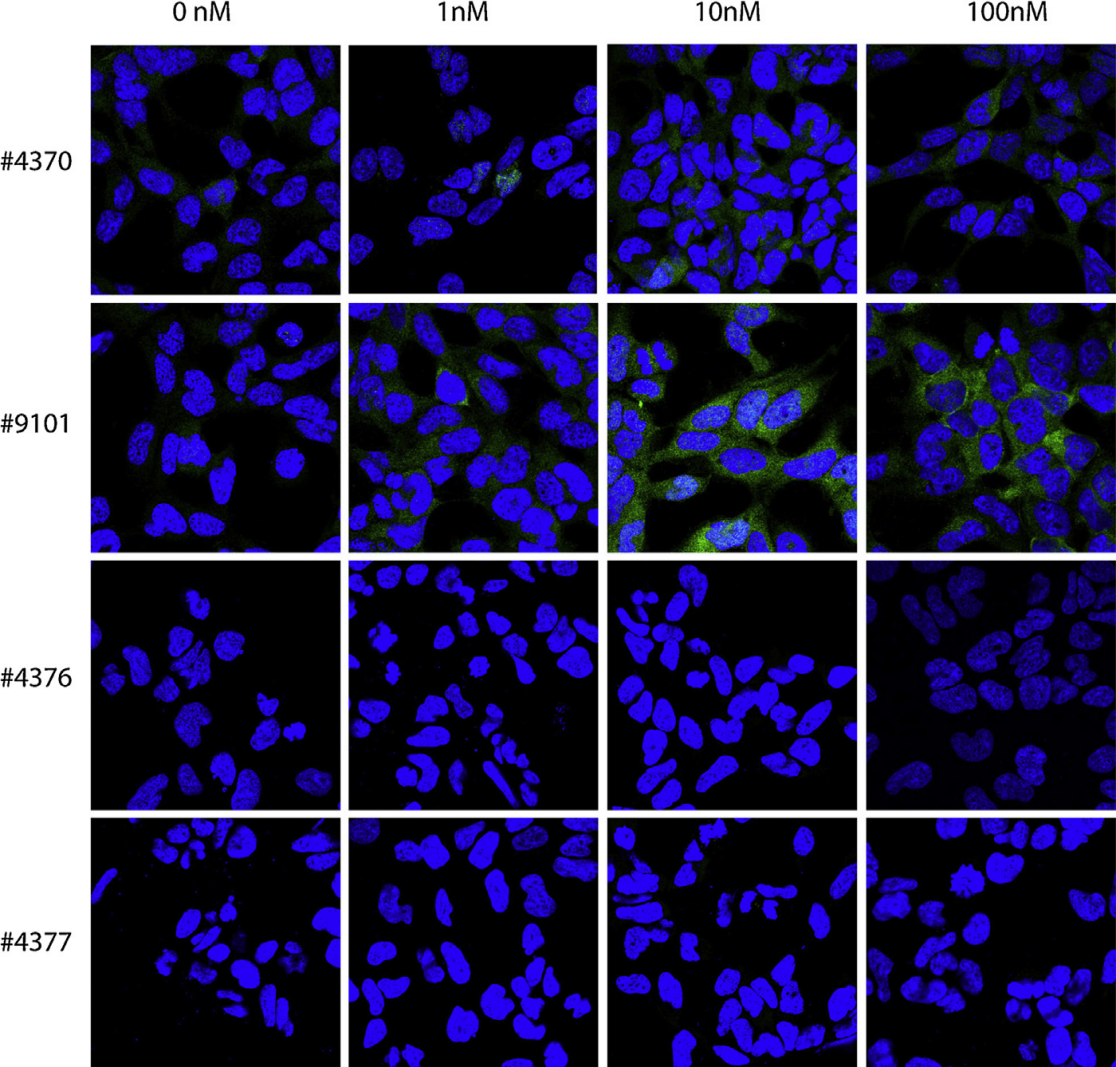
Immunocytochemical stainings against pMAPK of 0, 1, 10 or 100 nM insulin-stimulated HEK 293 cells permeabilized with Saponin. Out of the four different antibodies tested against pMAPK (#4370, #9101, #4376 and #4377), only #9101 and #4370 showed weak increase in signals upon stimulation with insulin compared to non-stimulated cells. The antibodies were tested in three independent experiments.

**Fig. 3:**
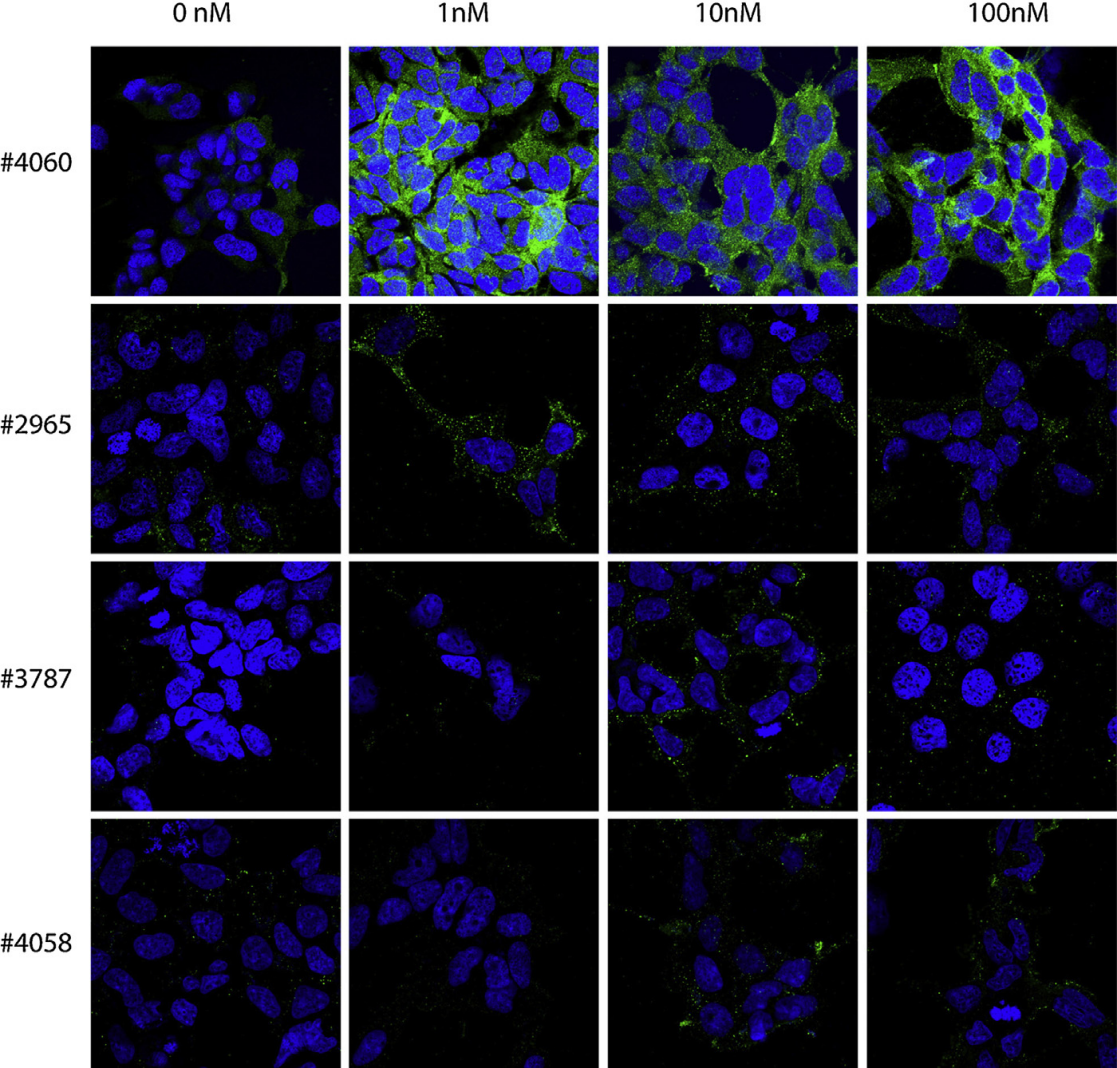
Immunocytochemical stainings against pAkt of 0, 1, 10 or 100 nM insulin-stimulated HEK 293 cells permeabilized with Triton X-100. Here, #4060 anti-pAkt antibody showed the most specific and strongest staining of pAkt compared with #2965, #3787 and #4058. The antibodies were tested in three independent experiments.

**Fig. 4:**
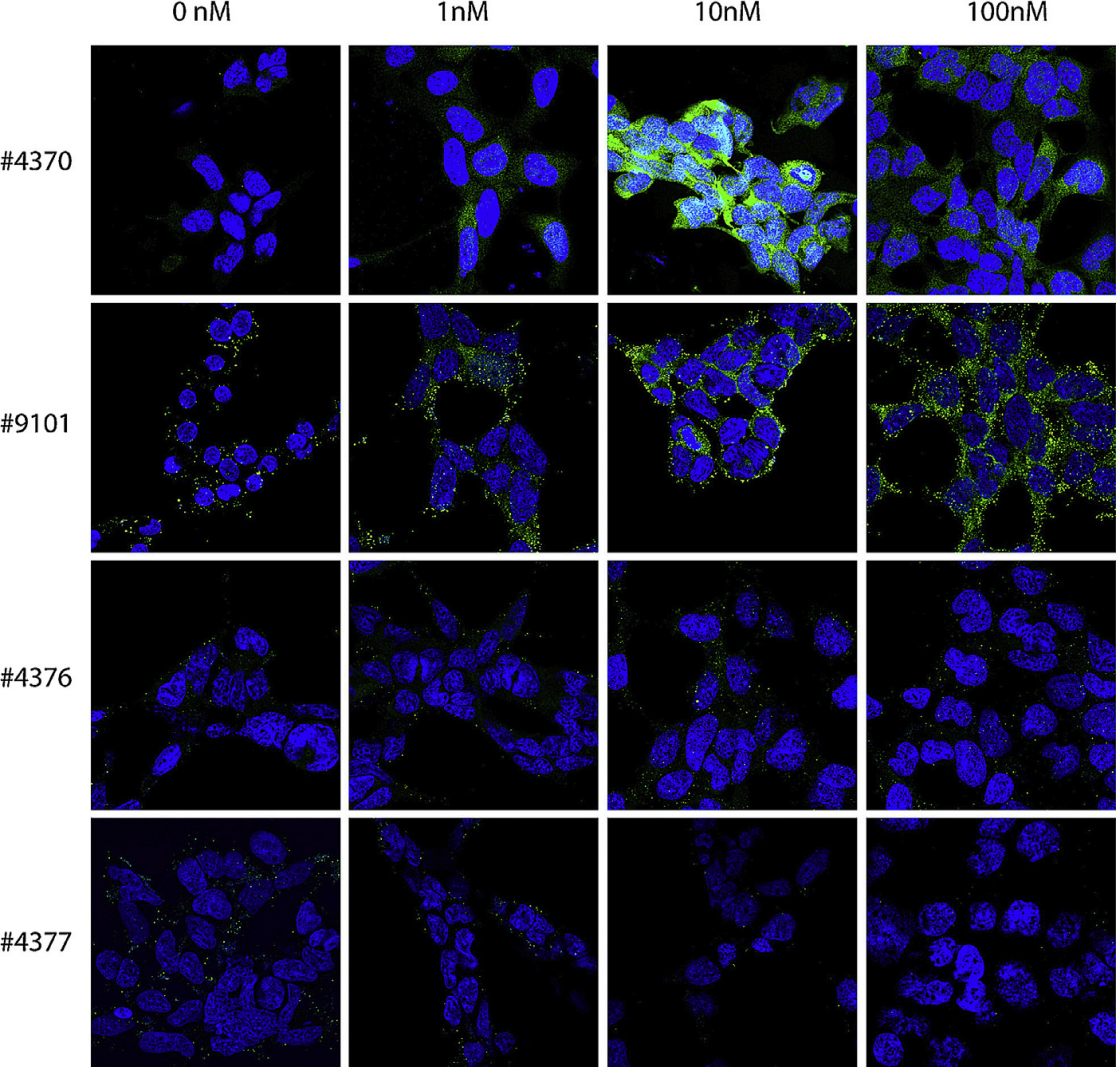
Immunocytochemical stainings against pMAPK of 0, 1, 10 or 100 nM insulin-stimulated HEK 293 cells permeabilized with Triton X-100. Using #4370 or #9101, a clear signal was seen upon stimulation compared to control. This was not the case when using #4370 or #4377. The antibodies were tested in three independent experiments.

Four different antibodies against pAkt and four different antibodies against pMAPK were initially tested on insulin-stimulated HEK 293 cells, and the two best antibodies against pAkt and pMAPK were then tested on BDNF-stimulated hippocampal neurons. The cells were stimulated with 1, 10 or 100 nM insulin (HEK 293 cells) or 1 or 10 nM BDNF (hippocampal neurons) or left unstimulated (medium without insulin/BDNF). When validating the antibodies, two different permeabilization agents were used; saponin (Fig. 1, Fig. 2) or Triton X-100 (Fig. 3, Fig. 4).

When using saponin as a permeabilization agent, none of the antibodies tested gave high levels of staining in cells stimulated with insulin (Fig. 1, Fig. 2). A weak signal was, however, observed with #9101 and #4370 (Cell Signaling) when staining for pMAPK (Fig. 2).

When cells were permeabilized with Triton X-100 instead of saponin, the stainings obtained against pAkt (Fig. 3) and pMAPK (Fig. 4) were markedly improved.

Staining against pAkt using #4060 or #2965 resulted in a clear signal in cells stimulated with insulin, compared to the signal obtained in unstimulated cells, as seen in Fig. 3.

**Fig. 5:**
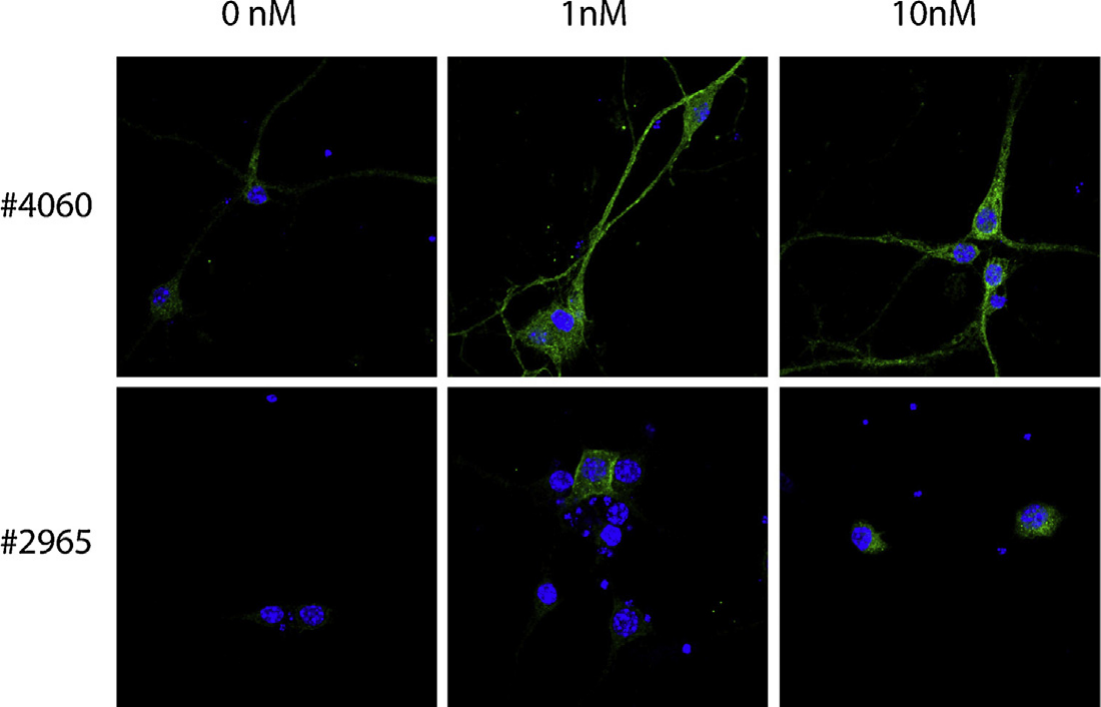
Immunocytochemical stainings against pAkt of 0, 1 or 10 nM BDNF-stimulated hippocampal neurons permeabilized with Triton X-100. The two antibodies against pAkt, #4060 and #2965, gave the best staining of HEK 293 cells and was therefore tested on hippocampal neurons. In three independent experiments, the #4060 antibody gave the strongest and most specific staining of hippocampal neurons.

**Fig. 6:**
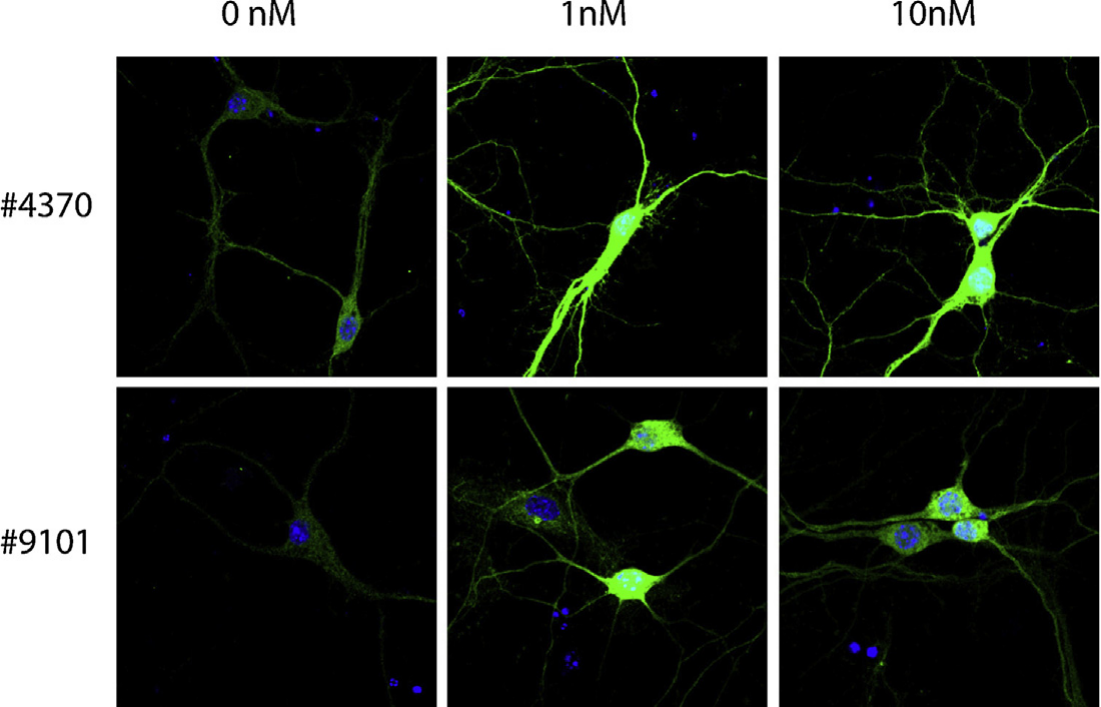
Immunocytochemical stainings against pMAPK of 0, 1 or 10 nM BDNF-stimulated hippocampal neurons permeabilized with Triton X-100. The two antibodies against pMAPK; #4370 and #9101 gave the best staining of HEK 293 cells and were therefore tested on hippocampal neurons. Here, the #4370 antibody gave the strongest and most specific staining of hippocampal neurons. The antibodies were tested in three independent experiments.

The difference in immunostaining intensities between insulin stimulated HEK 293 cells and non-stimulated cells were not as convincing when using the #3787 and the #4058 anti-pAkt (Cell Signaling), which indicates weaker binding of the antibodies to phosphorylated motifs, as compared to the #4060 and #2965 antibodies. ICC staining of BDNF-stimulated hippocampal neurons permeabilized with Triton X-100 also gave a clear signal when using the #4060 and #2965 anti-pAkt antibodies (Fig. 5). Thus, the anti-pAkt antibodies #4060 and #2965 are very suitable for ICC stainings of stimulated HEK 293 cells and hippocampal neurons permeabilized with Triton X-100. Likewise, permeabilization with Triton X-100 (Fig. 4) is more effective than permeabilization with saponin in order to obtain high-intensity staining levels, with low background staining when using anti-pMAPK antibodies in HEK 293 cells. The antibodies #4370 or #9101 proved better than #4377 or #4376, as they gave a stronger staining signal in the insulin-stimulated HEK 293 cells, compared to the unstimulated cells when permeabilizing the cells with Triton X-100. Especially #4370, but also #9101, were very suitable in detecting phosphorylation in BDNF-stimulated hippocampal neurons permeabilized with Triton X-100 (Fig. 6), as a high intensity and low background staining was observed in stimulated neurons, but not in unstimulated neurons.

**Fig. 7:**
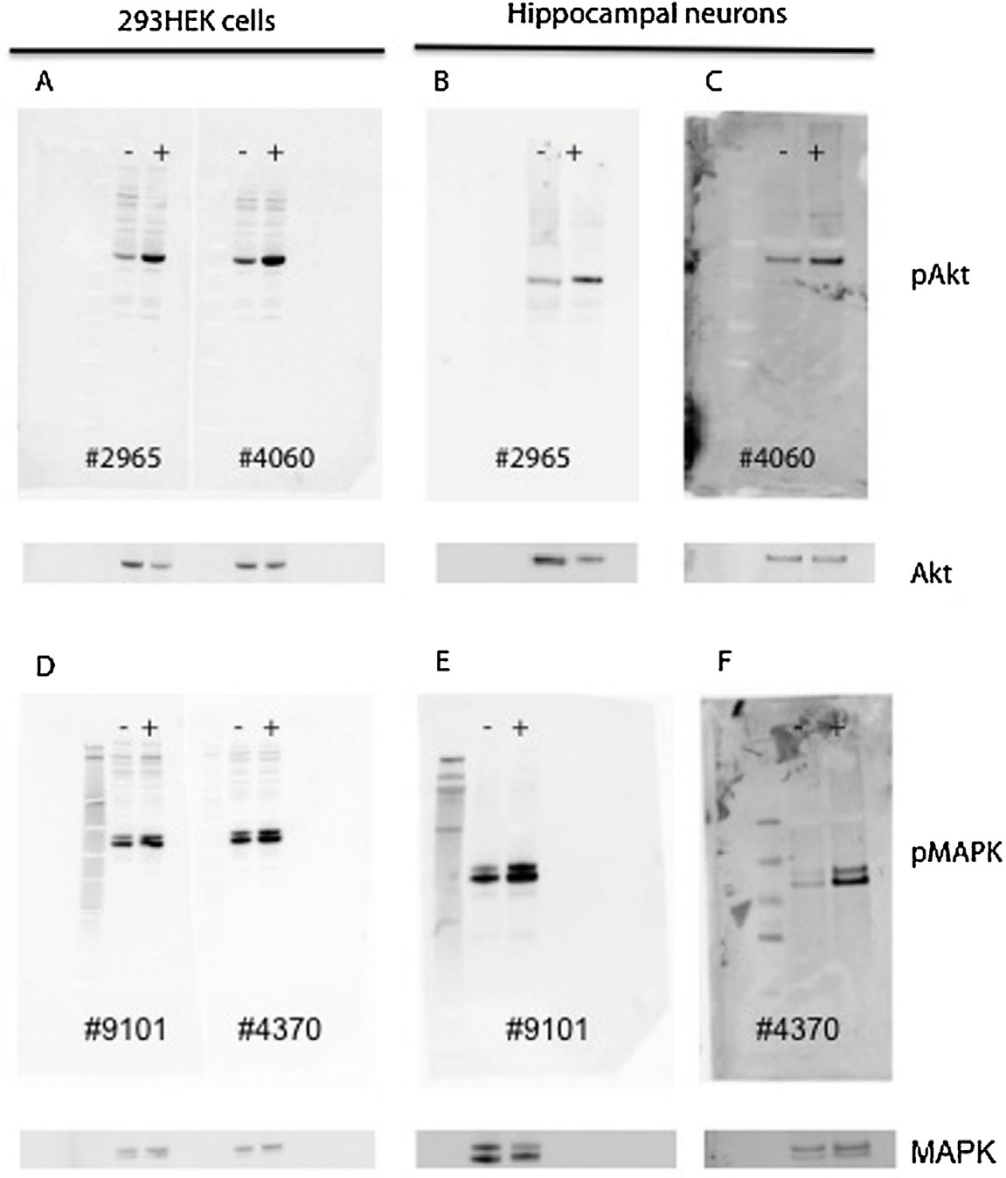
Western blot analysis. To validate the specificity of the antibodies, western blot analysis was performed with the two best antibodies against pAkt (#4060 and #2965) and pMAPK (#4370 and 9101) on lysate from HEK 293 cells, which had been stimulated with insulin (10 nM) and lysate from hippocampal neurons, which had been stimulated with BDNF (1 nM). Clear bands with the correct size for both pAkt (A–C, upper panel) and pMAPK (D–F upper panel) were detected with the used antibodies. The antibodies were able to detect a significant increase of the phosphorylated proteins after stimulation, observed as strong increases in band intensity between unstimulated (−) samples and stimulated (+) samples. To ensure that the increases in band intensities after stimulation were not due to different levels of Akt and MAPK, the membranes were stripped and visualized for total amount of Akt (A–C, lower panel) and MAPK (E–F, lower panel), showing that the observed increases in pAkt and pMAKP were indeed due to insulin/BDNF stimulation. The lanes to the left in figure D–F are molecular weight makers.

To confirm the specificity of the two best anti-pAkt antibodies and the two best anti-pMAPK antibodies, we performed western blot analysis on lysate from HEK 293 cells that had been stimulated with insulin and lysates from hippocampal neurons, where the neurons had been stimulated with BDNF. After visualizing for pAkt and pMAPK, the membranes were stripped and the total amounts of Akt and MAPK were investigated. This ensured that the observed increases in pAkt and pMAPK after stimulation were due to the stimulation and not due to different levels of total level of Akt or MAPK (Fig. 7).

As observed in Fig. 7A–C (upper panel), both antibodies against pAkt (#2965 and #4060, Cell Signaling) showed clear bands with correct band size, and increase in band intensity after stimulation with insulin or BDNF. Although some weaker bands with higher molecular weight were observed in the western blot with lysate from HEK 293 cells, these bands were not affected by insulin stimulation and were drastically lower in intensity compared to the bands with correct molecular weight. The observed increase in pAkt after stimulation was not caused by a higher level of total amount of Akt in the stimulated samples (Fig. 7A–C, lower panel).

For pMAPK, the results were similar to the ones observed for pAkt. Both insulin and BDNF caused a significant increase in band intensity with correct molecular weight compared to the band intensities observed in non-stimulated samples (Fig. 7D–F, upper panel). The observed increases in pMAPK were not due to difference in total amount of MAPK (Fig. 7D–F, lower panel). Moreover, similar to pAkt, bands with higher molecular weight than the ones for pMAPK were observed, however these bands were very weak, and band intensities were not dependent on stimulation.

In conclusion, this ICC protocol provides the parameters needed for successful detection of phosphorylated forms of kinases when using Triton X-100 as permeabilization agent and the #4060 and the #2965 anti-pAkt antibodies (Cell Signaling) or #4370 and #9101 anti-pMAPK antibodies (Cell Signaling). These antibodies were also found to perform well in western blotting. To help our fellow researchers we have rated the antibodies on the independent antibody-review site www.pAbmAbs.com in accordance with our findings (see Table 1 below).

This article highlights the importance of testing antibodies under different conditions. The antibodies used in this article have been tested at the concentrations suggested by the manufacturer. Other results might have been obtained if different concentrations of antibodies were tested. Moreover, formaldehyde fixation, which cross-links proteins, is used in this protocol. Formaldehyde might mask epiptopes and the antibodies might perform differently if MeOH, which denatures proteins, is used as the fixative, as the antibodies are made against synthetic phosphopeptides and potential epiptope masking is avoided

## Figures and Tables

**Table 1:** List of primary antibodies against pAkt and pMAPK used in this paper.

Antibody	Host	Clonality	Immunogen	Dilution factor	Company	Catalog nr.	Average pAbmAbs rating [1], [2], [3], [4], [5]
Anti-phospho-Akt (Ser473)	Rabbit	Monoclonal	Synthetic phosphopeptide corresponding to residues around Ser473 of human Akt	1:200	Cell Signaling	4060	
Anti-phospho-Akt (Thr308)	Rabbit	Monoclonal	Synthetic phosphopeptide corresponding to residues around Thr308 of mouse Akt	1:800	Cell Signaling	2965	
Anti-phospho-Akt (Ser473)	Rabbit	Monoclonal	Synthetic phosphopeptide corresponding to residues around Ser473 of mouse Akt	1:200	Cell Signaling	4058	
Anti-phospho-Akt (Ser473)	Rabbit	Monoclonal	Synthetic phosphopeptide corresponding to residues around Ser473 of mouse Akt	1:50	Cell Signaling	3783	
Anti-Akt	Rabbit	Polyclonal	Synthetic peptide corresponding to the carboxy-terminal sequence of mouse Akt	1:1000	Cell Signaling	9271	
Anti-phospho-MAPK (p44/42)	Rabbit	Monoclonal	Synthetic phosphopeptide corresponding to residues around Thr202/Tyr204 of human p44 MAP kinase	1:100	Cell Signaling	4376	
Anti-phospho-MAPK (p44/42)	Rabbit	Monoclonal	Synthetic phosphopeptide corresponding to residues surrounding Thr202/Tyr204 of human p44 MAP kinase	1:200	Cell Signaling	4377	
Anti-phospho-MAPK (p44/42)	Rabbit	Polyclonal	Synthetic phosphopeptide corresponding to residues surrounding Thr202/Tyr204 of human p44 MAP kinase	1:1000	Cell Signaling	9101	
Anti-phospho-MAPK (p44/42)	Rabbit	Monoclonal	Synthetic phosphopeptide corresponding to residues surrounding Thr202/Tyr204 of human p44 MAP kinase	1:200	Cell Signaling	4370	
Anti-MAPK	Rabbit	Polyclonal	Synthetic peptide corresponding to residues near the C-terminus of rat p44 MAP kinase	1:1000	Cell Signaling	4695	
